# Comparison of post-traumatic changes in circulating and bone marrow leukocytes between BALB/c and CD-1 mouse strains

**DOI:** 10.1371/journal.pone.0222594

**Published:** 2019-09-17

**Authors:** Tanja Spenlingwimmer, Johannes Zipperle, Mohammad Jafarmadar, Marcin Filip Osuchowski, Susanne Drechsler

**Affiliations:** Ludwig Boltzmann Institute for Experimental and Clinical Traumatology in the AUVA Research Center, Vienna, Austria; Heidelberg University Hospital, GERMANY

## Abstract

This manuscript emerged from a larger third-party funded project investigating a new poly-trauma model and its influence upon secondary sepsis. The present sub-study compared selected leukocyte subpopulations in the circulation and bone marrow after polytrauma in BALB/c versus CD-1 mice. Animals underwent unilateral femur fracture, splenectomy and hemorrhagic shock. We collected blood and bone marrow for flow cytometry analysis at 24h and 48h post-trauma. Circulating granulocytes (Ly6G+CD11+) increased in both strains after trauma. Only in BALB/c mice circulating CD8+ T-lymphocytes decreased within 48h by 30%. Regulatory T-cells (T_regs_, CD4+CD25+CD127^low^) increased in both strains by approx. 32%. Circulating Tregs and lymphocytes (CD11b-Ly6G-MHC-2+) were always at least 1.5-fold higher in BALB/c, while the bone marrow MHC-2 expression decreased in CD-1 mice (p<0.05). Overall, immune responses to polytrauma were similar in both strains. Additionally, BALB/c expressed higher level of circulating regulatory T-cells and MHC-2-positive lymphocytes compared to CD-1 mice.

## Introduction

Each year, approximately 11 million laboratory animals are used in Europe [1]. Mice account for approximately 70% of all animals used in preclinical research including critical care medicine making them the most frequently used species [2]. Generally, inbred strains such as BALB/c and C57BL/6 belong to the most frequently utilized [3]. There are strong arguments in favor of inbred strains; their relatively stable genetic uniformity [4] improves experimental reproducibility simultaneously increasing statistical power and reducing the total number of animals (the 3R rule). Additionally, the massive amount of genetic information available for inbred strains eases genetic manipulations and facilitates selection of mice with exact genetic characteristics desired for a defined experiment [5]. In toxicity testing, the use of small cohorts of inbred strains has been recently recommended [4,5]. In the last years, however, the importance of using genetically heterogeneous organisms in experiments has been stressed given that they better mimic the heterozygosity of the human population. The Diversity Outbred and Collaborative Cross mice initiative is an example of this attention shift [6]. The use of outbred mice allows a better insight into whether and/or to what extent the genotype influences the host response to disease. Outbred mice are commonly used in studies investigating malignancy and immunological responses to pathogens and/or drugs, aimed at recapitulating the diversity of human reactions to diseases and medications used to treat them [7–10].

Not many evaluations between inbred and outbred mouse strains were performed; the existing studies investigated conditions such as diabetes, bone marrow transplantation and obesity [11–16]. The vast majority of pre-clinical mouse studies on trauma were performed in inbred strains [12,17,18] and an available inter-strain comparison was performed among the inbred strains only [19].

Best to our knowledge, only two inbred versus outbred comparisons were performed in acute trauma models [20,21]. Another three [16,20,22] from the broad critical care field compared inbred to outbred mice demonstrating marked inter-strain differences in the investigated endpoints. For example, Richardson and Kuhn [16] demonstrated that inbred mice (compared to outbred) were typically more heterogeneous in their reactions to a cholera enterotoxin stimulus; several inbred strains (e.g. C3H, CBA, DBA) were less responsive than outbred (e.g. Swiss-Webster (CFW) strains. Cahill et al. [22] demonstrated that permeability of the blood-spinal cord barrier after severe peripheral nerve injury was approximately two-fold higher in CD-1 compared to BALB/c mice.

The above studies indicate that differences in immuno-inflammatory sequelae between the outbred and inbred strains are likely and should be accounted for in the context of study design in trauma modeling. Therefore, in a prequel to a larger study that investigated an influence of a new polytrauma model upon secondary sepsis [23], we compared the inbred and outbred mouse strains. Hence, this is not a stand-alone experiment, but it was designed in conjunction with the above project.

In the present study, we investigated whether the genetic hetero- and homogeneity of BALB/c vs. CD-1 mice subjected to polytrauma influenced subsequent immunological responses of selected leukocyte subpopulations in the circulating and bone marrow leukocyte compartment.

## Material and methods

### Animals

12 weeks old, female BALB/c (n = 60) and CD-1 (n = 33) mice (Charles River Laboratories, Sulzfeld, Germany) were used for the experiment, the exact n/group distributions are shown in the figure legends. Upon arrival all animals were allowed to acclimatize to their new environment for at least 7 days. Mice were kept in groups of 5 per type III cage, housed under a 12h light-dark diurnal cycle with a controlled temperature of 22°C to 24°C. Standard rodent diet and water were provided *ad libitum* throughout the experiment. Enrichment was provided (houses, wooden boards, wood wool and tissues for nesting) to facilitate natural behavior.

### Ethical statement

All animal procedures were approved by the Viennese (Austria) legislative committee (Animal Use Proposal Permission No. 343130/2013/14) and conducted according to the National Institutes of Health guidelines. These experiments were not stand-alone but aimed at selecting the most suited mouse strain for a large third-party funded research project [23]. The comparison of the immune responses to polytrauma between inbred and outbred mouse strains provided the basis to investigate the effect of the post-traumatic immunologic changes in the large project.

During the experiment mice were monitored by trained professionals several times a day to ensure their well-being, combining the observation of clinical symptoms with measurements of body weight and body temperature. Body weight and temperature remained within the range of the guidelines set by the Viennese legislative committee. All experiments were sublethal. Animals were euthanized by using deep inhalation anesthesia with isoflurane followed by cervical dislocation.

All surgical procedures were executed under inhalation anesthesia with isoflurane (2–3%, Forane®, Baxter, Austria). Analgesia (buprenorphine, 0.05 mg/kg, s.c., Bupaq®, Richter Pharma, Austria) was administered twice a day until the end of the experiment. Pre-existing baseline data from healthy BALB/c mice were included to reduce the number of animals to a minimum.

### Experimental design

BALB/c and CD-1 mice (in n = 10 per group iterations) were subjected to trauma (unilateral femur fracture), splenectomy and hemorrhagic shock (TSH). Either 24h or 48h later animals were euthanized to collect blood and bone marrow. The 24h and 48h time-points were chosen to detect protracted immunologic deterioration that can potentially influence the progression of (secondary) sepsis.

### Polytrauma model (TSH)

Trauma was induced by a closed unilateral mid-shaft fracture of the left femur with custom built blunt pliers, followed by hemorrhagic shock induced by a loss of 30% of total blood volume (calculated as 6% of the body weight) via retroorbital puncture under additional local anaesthesia (Oxybuprocainhydrochlorid, Novain®, AGEPHA Pharma, Austria).

Splenectomy was performed as follows: the left side of the abdomen was shaved and disinfected using betaisodona® solution. To open skin and abdominal cavity a 5mm incision was made. The spleen was carefully exposed. Before the removal of the spleen afferent and efferent blood vessels were ligated using Silkam® USP 4/0. Abdomen was closed with single button sutures and the skin using the Histoacryl® adhesive. Mice were resuscitated subcutaneously with four times of the shed blood volume, ¼ immediately post TSH, the remaining ¾ 1h post-TSH. All surgical procedures and the post-traumatic phase were covered with potent analgesia (0.05 mg/kg buprenorphine s.c., Bupaq®, Richter Pharma, Austria, twice daily until the end of the experiment).

### Blood samples

The first blood sample was taken at induction of trauma from shed blood (retroorbital puncture), the second one was collected via vena cava with a syringe rinsed with K_3_EDTA either 24h or 48h post-TSH.

### Bone marrow collection

Bone marrow was collected from the right femur and tibia. The epiphyses were cut off and the bones were flushed with 3ml cold PBS using a 23G needle. Collected bone marrow was gently pipetted through a 45 μm cell strainer and spun down at 300 x g for 10 mins. The pellet was resuspended in 2 mL lysis buffer (multispecies RBC lysis buffer, Thermo Fisher ebioscience, Waltham, MA, USA), incubated for 10 mins and neutralized by the addition of 2 mL PBS. After 2 washing steps, the pellet was resuspended in PBS and stained with fluorophore-labelled antibodies as described in the flow cytometry section. Flow cytometry was carried out using the same setup as for peripheral leukocytes.

### Flow cytometry analysis

The leukocyte markers used in this pre-clinical study were selected to match the markers that are commonly utilized to (immunologically) characterize polytrauma patients [24–27]; their selection also overlaps with corresponding animal studies [28–30].

Fluorescence activated cell sorting (FACS) was performed on a FC500 flow cytometer (Beckman Coulter Life Sciences, United States of America). The software used for visualization and analysis of the data was RXP (Beckman Coulter Life Sciences, USA).

EDTA-anti-coagulated blood was diluted 1:2 with PBS and stained native before lysis to examine the cellular immune status of the animals. Fluorophore-labelled anti-mouse antibodies against CD11b (FITC-conjugated), Ly6-G (PE-Cy5-conjugated), MHC-II (PE-conjugated), CD4 (FITC-conjugated), CD8a (PE-Cy7-conjugated), CD127 (PE-conjugated) and CD25 (PE-Cy5-conjugated) were purchased from eBioscience (Thermo Fisher, Waltham, MA, USA), antibodies and samples were prepared according to the manufacturers guidelines. Subsets of leukocytes were first identified by their morphological appearance (FSC-SSC) and in a second step through positivity or negativity for the specific surface markers. Granulocytes were defined as CD11b+Ly6G^high^-SSC^high^, monocytes as CD11+Ly6G^low^-SSC^low^. The expression of CD11b and MHC-II on those two cell subsets was monitored through the median fluorescence intensity of the respective antigens. FSC-SSC^low^ cells were assigned as lymphocytes, CD4+CD25+CD127^low^ cells as regulatory T-cells. T-helper cells were labelled as CD4+ and cytotoxic T-cells as CD8+. A schematic figure of the gating strategy to identify these subsets is provided in the supplementary material (S1 and S2 Figs).

### Statistical analysis

All data sets were tested for normality before analysis. Abnormally distributed data were logarithmically transformed (Y = log(Y)) to achieve normal distribution if possible. Differences among the groups were evaluated using one-way ANOVA for normally distributed and Kruskal-Wallis test for non-parametric data. Bonferroni/Dunn's Multiple Comparison post-hoc tests were used for all figures to compare data in each strain at different time-points (i.e. control vs. 24h vs. 48h; each figure panel) and simultaneously between strains at corresponding time-points (e.g. 24h BALB/c vs. 24h CD-1). Significance level was set at p = 0.05.

Data variability between BALB/c and CD-1 mice was analyzed by variation coefficients for all investigated parameters, followed by student’s T-test to compare each time point.

All statistical tests were carried out using Graphpad (Prism, Software Inc., San Diego, USA)

## Results

### Similar CD11b and Ly6G expression in inbred and outbred mice after trauma

**Fig 1:**
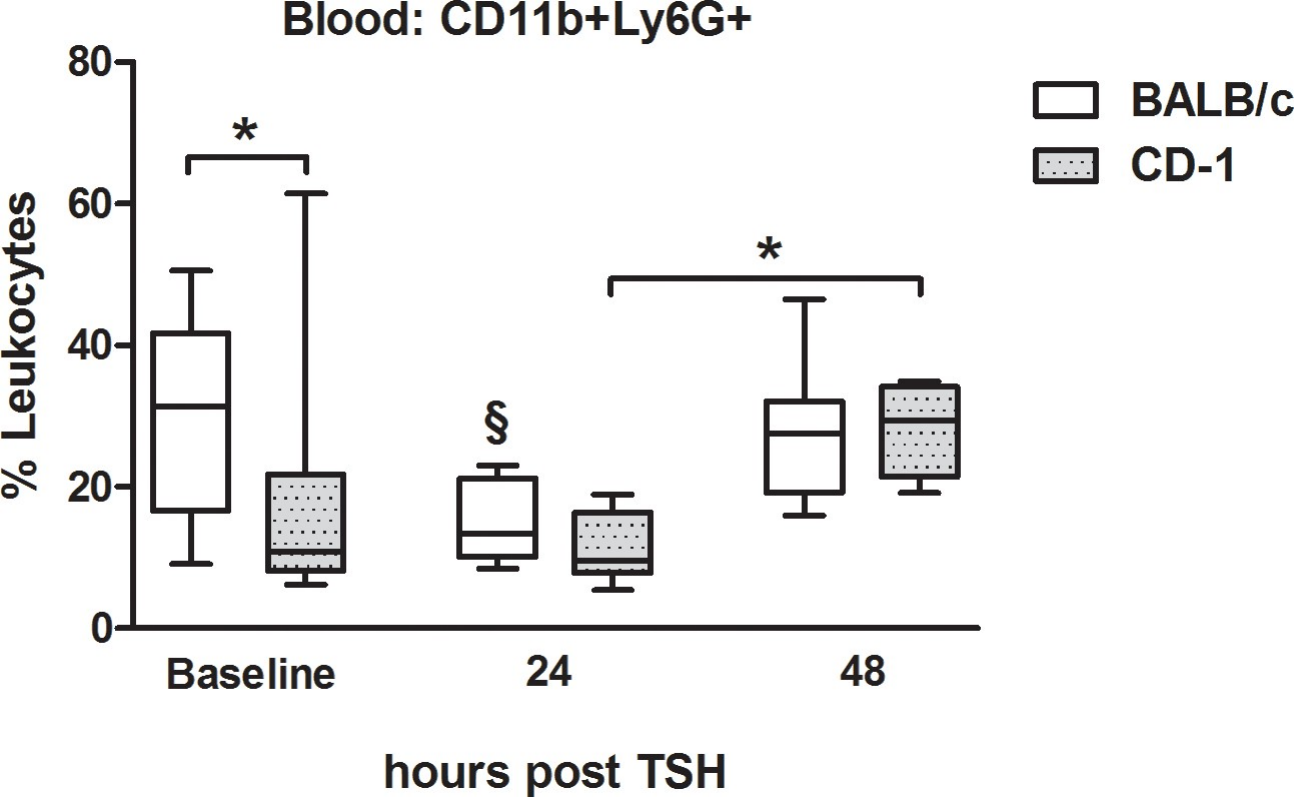
Post-traumatic changes of Ly6G+CD11b+ granulocytes in peripheral blood. 3-month-old, female BALB/c and CD-1 mice underwent TSH (femur fracture, splenectomy and hemorrhagic shock): n = 35 at baseline, n = 10 at 24h and n = 15 at 48h. CD-1 mice: n = 18 at baseline, n = 10 at 24h and n = 5 at 48h. *p<0.05; §p<0.05 compared to all other time points from the same mouse strain.

BALB/c and CD-1 mice showed an increase of activated granulocytes (Ly6G+CD11b+) from 24h to 48h after trauma (p<0.05), but only in BALB/c this was preceded by a transient drop at 24h compared to baseline (p<0.05) (Fig 1).

### Strain specific differences in CD4+CD25+CD127^low^ regulatory T-cells

**Fig 2:**
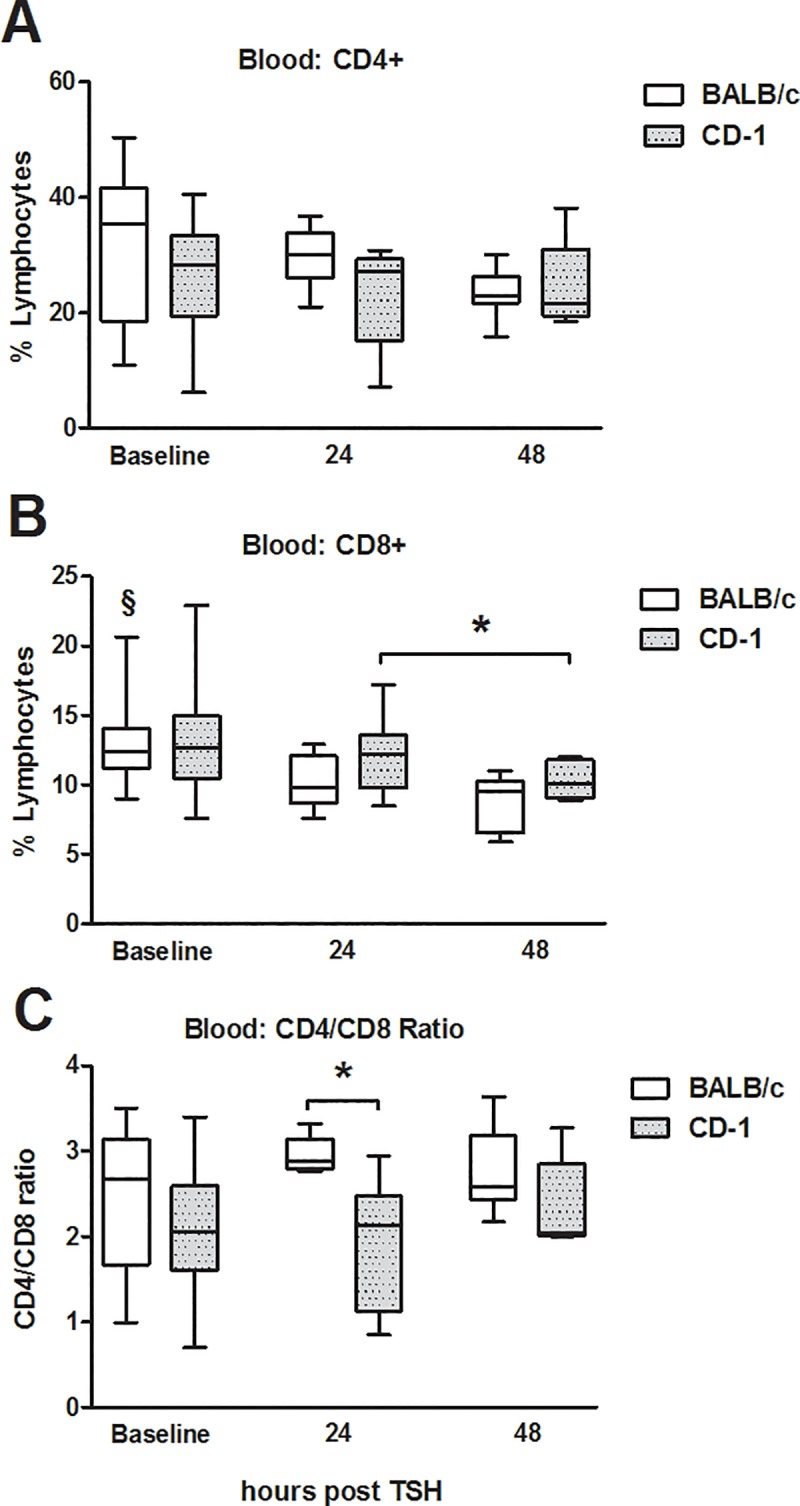
Post-traumatic alterations in circulating T-cell populations in peripheral blood. 3-month-old, female BALB/c and CD-1 mice underwent TSH (femur fracture, splenectomy and hemorrhagic shock): (A) CD4+ and (B) CD8+ T-cells (C) CD4/CD8 ratio. BALB/c mice: n = 35 at baseline, n = 10 at 24h and n = 15 at 48h. CD-1 mice: n = 18 at baseline, n = 10 at 24h and n = 5 at 48h. *p< 0.05.

**Fig 3:**
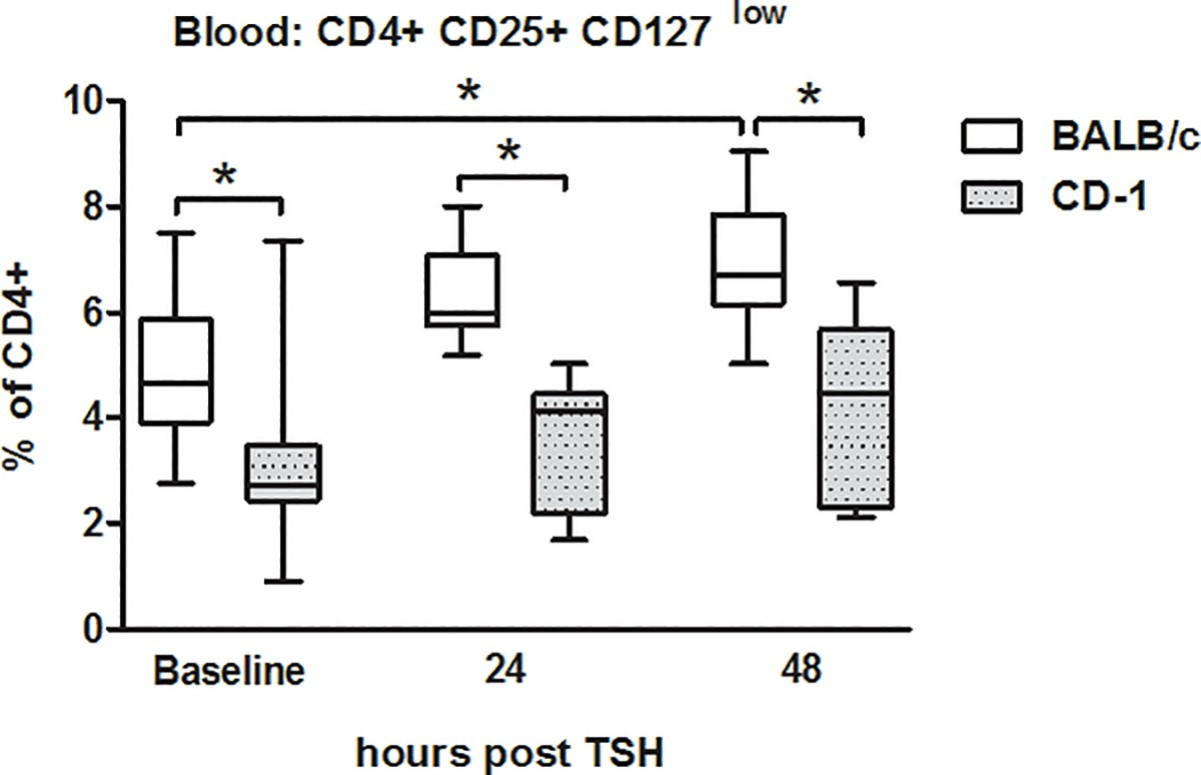
Post-traumatic alterations of CD4+CD25+CD127^low^ regulatory T-cells in peripheral blood. 3-month-old, female BALB/c and CD-1 mice underwent TSH (femur fracture, splenectomy and hemorrhagic shock). BALB/c mice: n = 35 at baseline, n = 10 at 24h and n = 15 at 48h. CD-1 mice: n = 18 at baseline, n = 10 at 24h and n = 5 at 48h. *p< 0.05.

Trauma induced a gradual decrease in CD4+ and CD8+ T-lymphocytes (Fig 2A and 2B) in both strains. This decrease was less pronounced in CD4+ T-cells from BALB/c mice, while their CD8-T-cells decreased by 20% and 30% at 24h and 48h compared to control (p<0.05). The CD4+/CD8+-ratio was increased in BALB/c mice compared to CD-1 at 24h after trauma (Fig 2C). Regulatory T-cells (CD4+CD25+CD127^low^) were at least 1.5-fold higher in BALB/c mice at all time points (p<0.05) (Fig 3). However, independent of the strain, polytrauma induced a gradual increase of circulating CD4+CD25+CD127^low^ cells over the 48h observation period; at 48h post-trauma, they were increased by 39% in BALB/c (p<0.05) and by 26% in CD-1 mice compared to baseline (Fig 3).

### Trauma-induced alterations in the blood and bone marrow lymphocytes in BALB/c and CD-1 mice

**Fig 4:**
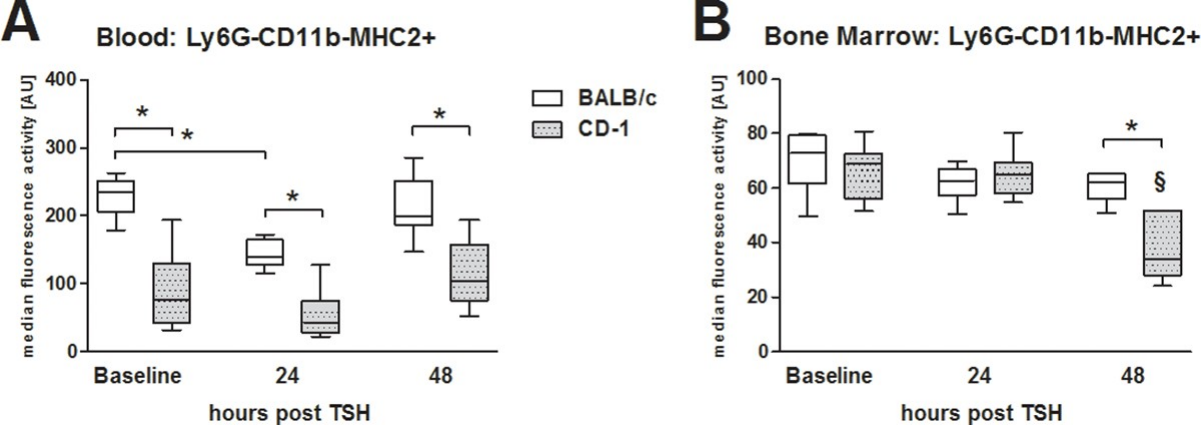
Alterations of MHC-2 expression on LY6G-CD11b- lymphocytes in the peripheral blood and the bone marrow. 3 month old, female BALB/c and CD-1 mice underwent TSH (femur fracture, splenectomy and hemorrhagic shock). (A) MHC-2 expression on lymphocytes in the peripheral blood, BALB/c mice: n = 35 at baseline, n = 10 at 24h and n = 15 at 48h. CD-1 mice: n = 18 in control (healthy), n = 10 at 24h and n = 5 at 48h. (B) MHC-2 expression on lymphocytes in the bone marrow. BALB/c mice: n = 10 at baseline, n = 10 at 24h and n = 5 at 48h. CD-1 mice: n = 10 in control (healthy), n = 9 at 24h and n = 5 at 48h. *p<0.05; ^§^p<0.05 compared to all other time points from the same mouse strain.

Trauma induced a transient 36% drop in CD11b-Ly6G-MHC-2+ lymphocytes isolated from the blood at 24h after trauma only in BALB/c, while in CD-1 mice they remained at baseline level (p<0.05). BALB/c mice had a continuously higher blood level of MHC-2-expressing lymphocytes (CD11b-Ly6G-MHC-2+) by at least 1.8-fold (Fig 4A). In the bone marrow of CD-1 mice, MHC-2 expression on lymphocytes decreased by 40% within 48h of trauma. However, BALB/c bone marrow lymphocytes did not respond to trauma; at 48h the level of CD11b-Ly6G-MHC-2+ cells was 1.5-fold higher than the one detected in CD-1 mice (Fig 4B).

### Less data variability in BALB/C mice at 24h

Data variability was analyzed using variation coefficients. BALB/c mice showed lower variability (0.15 vs. 0.38) of data at 24h after TSH (p<0.05). However, at baseline (control) and at 48h after trauma, variability was comparable between strains.

## Discussion

This (preliminary) study was conducted within the scope of a larger study with an overall aim to establish a composite poly-trauma hit including splenectomy and investigate its influence upon secondary sepsis. Based on the presented data (i.e. from the CD-1 versus BALB/C comparison), the BALB/c strain was selected for the main project [23]. We consider these comparative findings useful to researchers who employ mouse polytrauma models. Specifically, we subjected two of the most commonly used mouse strains, i.e. BALB/c (inbred) and CD-1 (outbred) to polytrauma (i.e. TSH) and compared selected leukocyte subpopulations in the blood and bone marrow compartments. The general trend in the posttraumatic leukocyte responses was similar between the two mouse strains; changes were only transiently more homogenous in BALB/c mice (i.e. at 24h post-TSH).

Polytrauma is one of the major causes of death in patients below 60 years [31,32]. If the severe injury itself is not immediately lethal, it can compromise the patients’ immune system predisposing them for complications such as secondary infections including sepsis. Numerous animal models have been employed over the years to experimentally recapitulate the most frequent clinical trauma sequelae including immunosuppression. Mice are within the widely accepted and preferably used model organisms to study immune reactions to such specific impacts due to their well-identified genetic background, the wide range of disposable surgical, technical and analytical methods, as well as their economic price. However, differences between the human and the murine immune/inflammatory response in the context of critical care medicine exist and have to be considered for an appropriate study design [33–36]. E.g. in contrast to humans, mice have a higher percentage of lymphocytes [37], they lack MHC-2 expression on T-cells [38,39] and CD4 expression on macrophages [40]. Furthermore, rodents need to be subjected to a much greater injury severity to elicit immune dysfunctions comparable to those occurring in human trauma patients [33,41]. That is why an effective preclinical modeling of the abovementioned scenario (i.e. posttraumatic phenotype of diminished immunocompetence) requires complex combinations of various injurious elements (e.g. hemorrhagic shock, fractures, chest trauma, cecotomy, traumatic brain injury). To date, only few preclinical studies compared immune or inflammatory responses between inbred and outbred mice in critical care conditions such as trauma and/or infection [16,21,22,42], thus definitive conclusions regarding the leukocyte dynamics are difficult to reach. In the studies by Rai et al. [21,42], intravenous injection of *Listeria monocytogenes* or *lymphocytic choriomeningitis virus (LCMV)* resulted in a higher variation in the magnitude of the CD8 T-cell response in outbred mice (Swiss Webster) independent of the infectious challenge [21]. In outbred mice, the memory CD8 T-cell pool-size and expansion rate of CD8+ T-cells were more heterogeneous and the magnitude between the effector response and infection dose was much more variable and did not correlate with the release of inflammatory cytokines [42]. In our study, polytrauma resulted in a decrease of CD8+ T-cells in both strains, however this change was significant only in BALB/c mice. Although the expression of CD4 on T-cells was comparable between inbred and outbred mice, we always detected an increased level in the subpopulation of regulatory T-cells (CD4+CD25+CD127^low^) in BALB/c mice. Much more pronounced differences in immune reactions between inbred and outbred mouse strains were demonstrated in a burn trauma study by Ma et al. [20]. First, thermally-induced mucosal damage containing predominantly lymphocytes and neutrophils could be shown in BALB/c, but not in CD-1 and C57/BL6 mice. Additionally, bacterial translocation across the gut mucosa occurred in 74% of BALB/c, but only in 7% of CD-1 mice without demonstrating any inter-strain differences in the cecal microbiome [20].

Of note, it appears that differences in the immune cell composition do not only occur between inbred and outbred strains but e.g. also within inbred strains. For example, Chen et al. compared CD4+CD25+ T_regs_ between BALB/c and B/6 mouse strains and observed higher T_regs_ counts in BALB/c mice [43]. Such differences between two inbred strains also occurred in the study from Matsutani et al. [44], who stimulated splenocytes and bone marrow cells from C3H/HeN and C57BL/6J mice (subjected to trauma and hemorrhage) with Concavalin A and demonstrated that the genetic background resulted in differences in the proliferation rates and the release of cytokines.

Variation coefficients were lower in BALB/c mice compared to CD-1 only at 24h after trauma, which could be due to their more uniform genetic background [22]. Inbred strains have heterozygous call rates greater than 2% [45]. In contrast, e.g. Charles River Laboratories guarantees 28–29% heterozygosis for CD-1 mice [46]. However, it can be presumed that the approximate 30% heterozygosis in outbred mice does not appropriately reflect the larger heterozygosis characteristic of the human population. Although a higher percentage of heterozygosis as seen in outbred mice more likely represents the genetic variations seen in the human population, the solely use of outbred mice in the present study would not have provided additional information.

## Conclusion

Our study showed that the immune responses to polytrauma were similar between inbred BALB/c and outbred CD-1 mice. BALB/c mice displayed a higher level of circulating regulatory T-cells and MHC-2-positive lymphocytes compared to CD-1 mice.

## Supporting information

S1 Fig **Representative gating strategy for leukocyte subsets with an emphasis on (A) Lymphocytes and (B) Monocytes.** Diluted peripheral whole blood was subjected to RBC lysis and divided into a Lymphocyte and Monocyte panel. Lymphocytes: A CD4+ population was gated from all captured events and further assessed for the positivity for CD25. Using fluorescence-minus-one (FMO) stainings, the respective population was then plotted against CD127 to identify a CD4+CD25+CD127- subset.
Monocytes: All captured events were analyzed for the presence of CD11b and Ly6G and were divided into CD11b+Ly6Ghigh, CD11b+Ly6Glow and CD11b-Ly6G-. The defined populations were then regated in dot blots with fluorescence channels for MHC-2 and CD11b versus side scatter (SSC). In addition, simultaneous stainings with CD11b and F4/80 were carried out to confirm gating based on CD11b signal versus SSC. Fluorescence from a specific antigen is given as mean fluorescence intensity (MFI) from the respective conjugate. Absolute counts of identified events were calculated per volume and given as events/μL.
(TIF)

S2 Fig Representative gating strategy for monocyte-derived macrophages and the polarization towards Arginase I and inducible NO synthase (iNOS) metabolism. Peripheral blood was stained with antibodies targeting extracellular antigens (CD11b, MHC-2 or Ly6G, CD4), fixed, permeabilized and subsequently stained for the intracellular antigens Arginase I and iNOS. Lymphocytes and Granulocytes were identified based on positivity/ negativity for CD4 and Ly6G and morphology in the FSC-SSC. A combinating gate with an exclusion logic for granulocytes was defined based on positivity for CD11b, MHC-2 and iNOS. The respective subpopulation was then plotted in an iNOS versus Arginase I window and the expansion of the population towards increased Arginase I or iNOS expression was defined as polarized activation.
(TIF)

S1 Table Experimental raw data generated by flow cytometry. (XLS)
