# Supplementary figures and images for: Comparison of post-traumatic changes in circulating and bone marrow leukocytes between BALB/c and CD-1 mouse strains
Source: PLoS One. 2019 Sep 17;14(9):e0222594. doi: 10.1371/journal.pone.0222594 (PMC6748677; doi:10.1371/journal.pone.0222594)

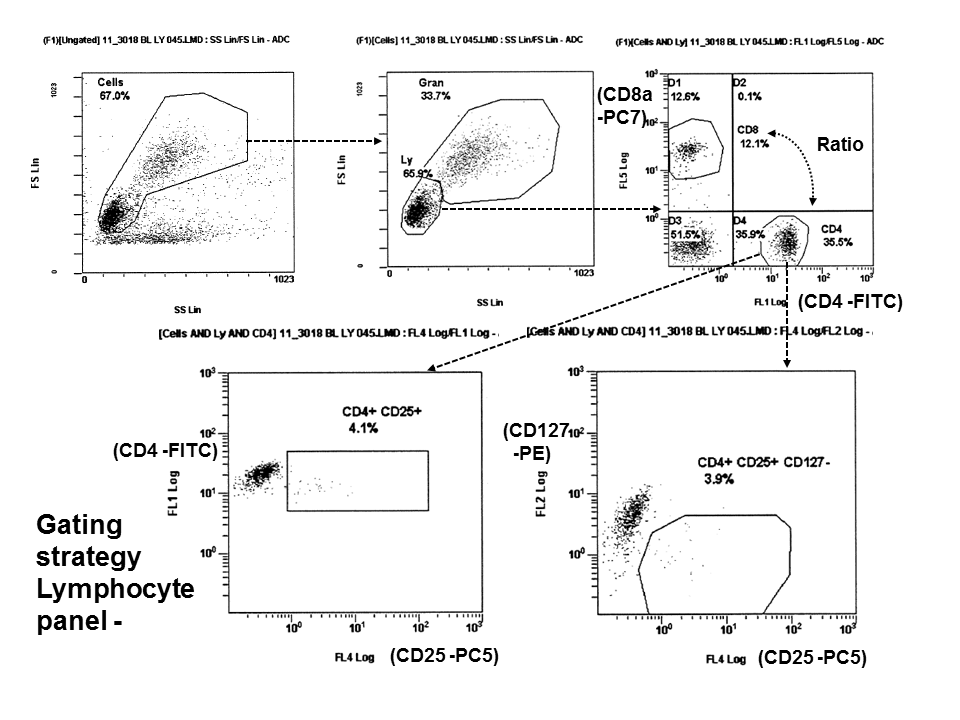

Supplement: S1 Fig — Representative gating strategy for leukocyte subsets with an emphasis on (A) Lymphocytes and (B) Monocytes. Diluted peripheral whole blood was subjected to RBC lysis and divided into a Lymphocyte and Monocyte panel. Lymphocytes: A CD4+ population was gated from all captured events and further assessed for the positivity for CD25. Using fluorescence-minus-one (FMO) stainings, the respective population was then plotted against CD127 to identify a CD4+CD25+CD127- subset. Monocytes: All captured events were analyzed for the presence of CD11b and Ly6G and were divided into CD11b+Ly6Ghigh, CD11b+Ly6Glow and CD11b-Ly6G-. The defined populations were then regated in dot blots with fluorescence channels for MHC-2 and CD11b versus side scatter (SSC). In addition, simultaneous stainings with CD11b and F4/80 were carried out to confirm gating based on CD11b signal versus SSC. Fluorescence from a specific antigen is given as mean fluorescence intensity (MFI) from the respective conjugate. Absolute counts of identified events were calculated per volume and given as events/μL. (TIF) [file pone.0222594.s001.tif]

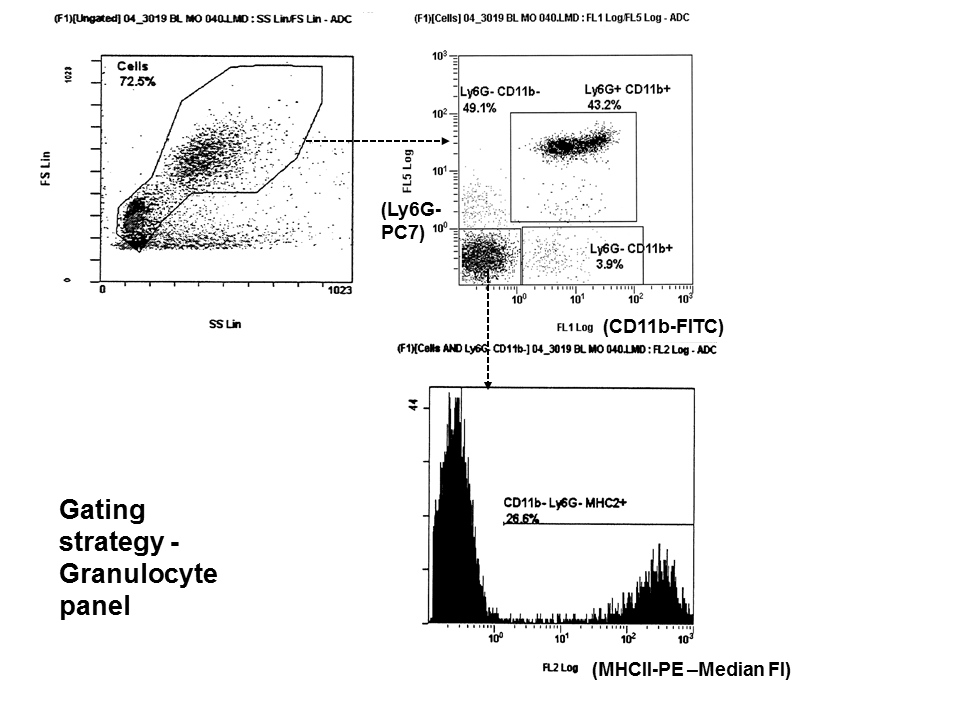

Supplement: S2 Fig — Peripheral blood was stained with antibodies targeting extracellular antigens (CD11b, MHC-2 or Ly6G, CD4), fixed, permeabilized and subsequently stained for the intracellular antigens Arginase I and iNOS. Lymphocytes and Granulocytes were identified based on positivity/ negativity for CD4 and Ly6G and morphology in the FSC-SSC. A combinating gate with an exclusion logic for granulocytes was defined based on positivity for CD11b, MHC-2 and iNOS. The respective subpopulation was then plotted in an iNOS versus Arginase I window and the expansion of the population towards increased Arginase I or iNOS expression was defined as polarized activation. (TIF) [file pone.0222594.s002.tif]
